# Have researchers in health professions education pathologised medical training?

**DOI:** 10.1111/medu.70243

**Published:** 2026-05-15

**Authors:** Dorene F. Balmer, May Shum, A. Emiko Blalock

**Affiliations:** ^1^ Department of Pediatrics Perelman School of Medicine at the University of Pennsylvania Philadelphia Pennsylvania USA; ^2^ CHOP Education Collaboratory Children's Hospital of Philadelphia Philadelphia Pennsylvania USA; ^3^ Department of Family Medicine, College of Human Medicine Michigan State University East Lansing Michigan USA

## Abstract

Have researchers in health professions education pathologized medical training? Longitudinal qualitative research suggests that trainees are not flattened by training; they are transformed by it. #MedEd

Researchers in the field of health professions education typically approach scientific inquiry with a pragmatic lens, seeking to elucidate shared problems and, in turn, their solutions.^1^ In this issues of *Medical Education*, Pattinson, Savelberg and Atherley use longitudinal audio diaries to explore the ‘problem’ of transitioning from medical student to junior doctor.^2^ While that transition was indeed stressful, the authors note, ‘Challenges can be seen as opportunities for valuable personal and professional development where transformational experiences are lived and reflected on’. They call for a conceptual shift away from seeing transitions as discrete events, which health professions education almost invariably frames as problematic, and toward seeing them as ongoing, complex processes.

We endorse this frameshift. As longitudinal qualitative researchers, we are uniquely positioned to follow the trajectory of these ‘problems’ as they evolve over time. One of us interviewed 15 women throughout their 4 years of medical school to understand their process of becoming doctors within a gendered organisation.^3^ Another interviewed six medical students as they became residents, fellows and ultimately practicing physicians over the course of 12 years, exploring the challenges they faced when constructing a career in medicine or surgery.^4^ Having the privilege of sustained engagement with participants in these studies, both of us found ourselves asking, has health professions education pathologised medical training? Have we made training a condition that needs to be treated, if not cured?

Having the privilege of sustained engagement with participants in these studies, both of us found ourselves asking, has health professions education pathologised medical training?

Clearly, there are problems. For instance, suicide was the most prevalent cause of death for US medical residents and fellows from 2015 to 2021, a statistic that is wholly unacceptable.^5^ Moreover, the very structure of medical training is one rooted in exclusion and still struggles to recruit and support a diverse student body.^6^ In health profession education, researchers' problem‐based focus may suggest that the long arc of training inevitably bends toward depletion. In our experience, however, when trainees narrated their own growth as aspiring doctors, they viewed their paths in a much less pathological manner.

In our experience, however, when trainees narrated their own growth as aspiring doctors, they viewed their paths in a much less pathological manner.

Take empathy, for example. Research reporting a decline in empathy with clinical training may be interpreted as an inevitability for medical students.^7^ Aspiring doctors believed the research that foreshadowed an inevitable decline in empathy as training progressed; they worried that they would become *jaded*. But, to their own surprise, they did not. Rather, how they conceptualised empathy changed through time, in response to the challenges of medical training. When asked about earlier warnings that empathy would decline, one newly minted neonatologist pushed back: ‘I think if anything, I have more empathy’. She struggled with conceptualising her care as *empathy*, per se, recognising that she could not completely understand what someone else was feeling. But her commitment to empathic care did not decline: ‘In the context of [this hospitalization] being one of the worst things that ever happened to them, I'm just being good to them. And trying to help’. What we heard by listening to aspiring doctors across years of training was not simply a pathological decline in empathy but an evolving relationship with empathy: the complex pathophysiology of training.

What we heard by listening to aspiring doctors across years of training was not simply a pathological decline in empathy but an evolving relationship with empathy: the complex pathophysiology of training.

Focusing on problems might be at the core of the field of health professions education, but it is time to ask: What is at stake for trainees if we continue to pathologise training? As Thomas and Ellaway advise, considering everything as a problem restricts how we view training in health professions education.^8^ We take that assertion one step further, speaking from our experience with longitudinal research relationships with trainees. If we focus only on pathology and *what* is wrong, we lose sight of pathophysiology and *how* trainees respond to the highs, the lows and the in‐betweens of medical training. When problems dominate the discussion, medical trainees lean into how medical education has damaged them—or, as one participant said, ‘the trauma inherent to medical education’. What is missing is the longer view. Three years later, that same participant said this when asked to offer career advice to her younger self: ‘I would just tell myself to trust that your career will work out. No, not trust that it will work out but trust that YOU will make the best of what happens’.

What is at stake for trainees if we continue to pathologise training?

We argue that if researchers in health professions education considered sustained engagement with medical trainees, we would see the throughline of change, the ebbs and flows that roll quietly into transformation. Thus, we propose a shift in how we think about problems and encourage our research peers to consider the following:

- *Bear witness to the growth potential of trainees:* Rather than determining solutions for trainees, we can partner with them to identify their baseline and support the strategies and solutions they develop over time. Sustained engagement allows us to learn how they navigate the everyday and the critical events of learning to be a doctor.
- *Trace the interplay of the individual and interpersonal within the larger sociocultural context of medical training:* While honouring each trainee's starting point, we also have an opportunity—and perhaps an obligation—to connect their stories to the social, political, and economic forces shaping medical education. Longitudinal engagement enables us to zoom in on personal, often intimate accounts and then zoom out to understand how these accounts are shaped by the larger sociocultural landscape.
- *Foreground change and transformation as a process, not a product*: A longitudinal approach highlights transformation not as a fixed outcome, but as a continuous journey. This perspective allows us to celebrate diverse career trajectories rather than regretting those that diverge from predefined career paths.

If we continue to pathologise training, we may narrow the moral and imaginative space of their chosen profession. Trainees are not flattened by training; they are transformed by it—sometimes painfully, often imperfectly, but almost always meaningfully. As Pattinson, Savelbert and Atherley call out, challenge is not always a problem.^2^ Shifting our research perspectives to value longer engagement with trainees may help us recognise how they traverse the terrain of their learning. In doing so, we may be helping to make stories of flourishing and hope come true.

## AUTHOR CONTRIBUTIONS

**Dorene F. Balmer:** Conceptualization; writing—original draft; writing—review and editing. **May Shum:** Conceptualization; writing—original draft; writing—review and editing. **A. Emiko Blalock:** Conceptualization; writing—original draft; writing—review and editing.

## Data Availability

Data sharing is not applicable to this article as no datasets were generated or analysed during the current study.
